# Ethics, Evidence and Economics in the Pursuit of “Personalized Medicine”

**DOI:** 10.3390/jpm4020137

**Published:** 2014-03-27

**Authors:** Jan Lewis, Wendy Lipworth, Ian Kerridge

**Affiliations:** 1 Centre for Values Ethics and Law in Medicine, University of Sydney Medical Foundation Building (K25), University of Sydney, Sydney, NSW 2006, Australia; 2 Australian Institute of Health Innovation, University of New South Wales, Sydney, NSW 2052, Australia; E-Mail: wendylipworth@gmail.com; 3 Centre for Values, Ethics and Law in Medicine, University of Sydney Medical Foundation Building (K25), University of Sydney, Sydney, NSW 2006, Australia; E-Mail: ian.kerridge@sydney.edu.au

**Keywords:** individualized medicine, pharmacology, ethics, economics, medicine, knowledge

## Abstract

Despite enthusiastic advocacy for what personalized medicine might be able to deliver and major investments into the development of this, there remain disappointingly few examples of personalized medicine in routine clinical practice today, particularly in high areas of unmet need such as cancer. We believe that this is because personalized medicine challenges the moral, economic and epistemological foundations of medicine. In this article, we briefly describe the scientific premises underpinning personalized medicine, contrast these with traditional paradigms of drug development, and then consider the ethical, economic and epistemological implications of this approach to medicine.

## 1. Introduction

The phrase “personalized medicine” has become something of a “term of art”, which means different things to different people. One understanding of personalized medicine (PM) focuses on the incorporation of molecular insights into treatment selection. Evolution of this form of PM has become a possibility because molecular biology has yielded extraordinary insights into the origins of diseases such as cancer and other chronic illnesses. These have revealed both the enormous heterogeneity and the clinical significance of a number of disrupted biological processes associated with development and progression of disease. Some have been linked directly with underlying genetic alterations, arising either at the time of conception (germ-line mutations) or during the life of an individual (somatic mutations). These advances, in turn, have led to the hope that PM will enable further progress in disease prevention, diagnosis and the delivery of tailored therapies.

Yet, despite enthusiastic advocacy, major investments into the development of molecularly targeted agents and advances in diagnostic technologies, there remain few examples of these kinds of therapies in routine clinical practice today. It is therefore important to ask why progress in this form of PM may not (yet) have achieved its expected potential, and what might be standing in the way.

We believe that despite being framed as an unproblematic scientific advance, this form of PM in fact challenges both the epistemological foundations of medicine and a number of fundamental moral and economic principles. While not all the issues we will discuss are unique to PM based on molecular diagnosis, they are particularly salient in this context. In this article, we briefly describe the scientific premises underpinning PM, contrast these with traditional paradigms of drug development, and then consider the ethical, economic and epistemological implications of this approach to medicine.

## 2. Science of Personalized Medicine Based on Molecular Diagnosis

As with other “terms of art” a number of interpretations and definitions exist for PM, by the way it might both be practiced and what it seeks to achieve. Although the notion of patient-centric and individualized healthcare aligns well with the philosophy of PM, narrower contemporary definitions, such as the one crafted by a Harvard Medical and Harvard Business School meeting in 2006 typically specify “the management of a patient’s disease or disposition by using molecular knowledge to achieve the best possible medicinal outcome for that individual” [1]. This narrower interpretation of PM, which is the one we will use in this article, assumes that interactions between drugs and protein receptors lead to modulation of signaling pathways that are associated with the development, maintenance or progression of disease.

This “biological modulation” may involve a native physiological process (via what we will term *physiological* targeting) or an aberrant process that is ordinarily absent in a healthy state. The latter strategy we will refer to as *pathological* targeting. Examples of physiologically targeted treatments include statins and angiotensin-converting-enzyme (ACE) inhibitors. Examples of pathologically targeted treatments include antibiotics and tyrosine kinase inhibitors (TKIs) used in cancer management.

The effectiveness and toxicity profile of a drug (extrinsic biological factors) will depend upon the nature of interactions between the drug and, “on-” and “off-” target receptors (intrinsic biological factors). The nature of drug-receptor interactions (pharmacodynamic profile) and the drug’s movement into, within and out of the body (pharmacokinetic profile) will determine the duration and magnitude of effects and possible toxicities experienced by a patient. These pharmacodynamic (PD) and pharmacokinetic (PK) profiles may differ significantly between patients administered the same dose of drug due to genetic, physical, physiological and metabolic differences between individuals.

Thus, the practice of the form of PM based on molecular diagnostics would ideally recognize each patient’s unique PD and PK profile and attempt to tailor treatments for each individual. Identifying the presence of a pathological target is prerequisite for the implementation of a successful therapeutic strategy whereas the profiling of patients’ PD and PK profiles are critical to ensuring drug efficacy is not overshadowed by toxicity. 

## 3. Ethical Challenges of Personalized Medicine based on Molecular Diagnosis

For PM, or indeed any other scientific, clinical or policy development to be embraced, it should be congruent with the moral dimensions of healthcare, the broader needs and preferences of patients, and the obligations of practitioners and policymakers. While it is beyond the scope of this article to discuss the moral dimensions of PM in detail, bioethicists who have written about PM have been concerned primarily with the extent to which a PM based on molecular diagnosis: (1) advances patient autonomy and agency; (2) promotes wellbeing; (3) prevents or minimizes harm; and (4) enables fairness and equity. These are well-recognized principles of biomedical ethics, but they play out in particularly interesting ways in the context of this kind of PM.

At first glance it might seem that the premise of molecularly-targeted PM should easily align with the need to promote autonomy, do good and minimize harm. The acquisition of additional diagnostic knowledge for the purpose of predicting a positive treatment outcome, and the screening of patients for predictive molecular signatures associated with greater predisposition to drug toxicities, are fundamentally patient-centered approaches to healthcare. Both also provide additional information for decision-making, thus increasing the likelihood of benefit and decreasing the likelihood of harm. However the reality is more complicated as the potential also exists for a PM based on molecular diagnosis to both compromise patient autonomy and cause harm.

Threats to autonomy are likely to arise if, for example, public or private insurers begin to coerce patients into having genetic tests as a determinant of coverage of medicines. As Vogenberg *et al.* argue [2]:

*“Whether an individual’s right to autonomy is primary and, therefore, a greater ethical requirement than society’s right to maintain efficient and effective care, or a payer’s right to avoid unnecessary cost burdens, remains problematic and illustrates a core issue in integrating PM into clinical care. Who should control access to new pharmacogenetic and pharmacogenomic tests and companion diagnostics?”*

[2]

In addition to threatening patients’ autonomy, the genetic tests associated with molecular diagnoses for PM pose all of the risks associated with genetic testing more generally, most notably potential breaches of confidentiality and subsequent genetic discrimination [2]. Harm might also stem from fact that it can be difficult to establish the safety of PMs. For reasons that will be discussed in detail below, personalized medicines can be difficult to test in clinical trials, so the ethical issues surrounding the use of innovative health technologies, with their often unclear and unpredictable risk—benefit ratios [3] are likely to be particularly significant in this context.

With respect to justice, it might seem logical that strategies to facilitate selection of only those patients likely to benefit and/or experience lower toxicities would be a more cost effective approach compared to the empirical selection of treatments. The use of treatments that do not work wastes resources, potentially exposes patients to costly adverse-effects, and delays time to effective treatment. Therefore funding only these treatments that are likely to work (and only the necessary companion diagnostics) go some way towards reducing waste in health systems. Also, because targeted therapies might be the only therapeutic option for particular individuals or sub-populations, the development of new PMs based on molecular diagnoses might promote equity amongst those patients with limited or clinically suboptimal options.

However, this kind of personalized medicine poses a number of important challenges for those concerned with promoting justice, such as governments and insurance companies, who have to make decisions about distributing limited health care resources.

This is because, while some molecularly-targeted PM strategies may be “affordable” to those with sufficient means to pay for them, they are often expensive because companies want to recoup the costs associated with developing medicines that, by definition, are suitable for only a limited number of patients. Payers may, therefore, be asked to pay large sums of money for diagnostic tests and treatments that help only a small subset of the population. This is not necessarily a problem if these therapies are highly effective, but this is not always the case.

In these situations, funding medicines not only creates significant opportunity costs, but also has the potential to conflict with and potentially undermine funding systems based on population-level cost effectiveness models. This was clearly illustrated by the events that unfolded when Australia’s Pharmaceutical Benefits Advisory Committee (PBAC) recommended against including trastuzumab (Herceptin) for the treatment of metastatic breast cancer on the national formulary, on the basis that it was not cost-effective. Public and medical lobbying that followed this decision was such that the government was pressured to set up a special fund, outside of the national formulary, to fund Herceptin [4]. This was the first time that the usual government funding system had been circumvented, and it is noteworthy that this event was focused on one of the most highly touted targeted therapies.

It is also noteworthy that these events took place in a wealthy country, because threats to justice are even starker in low- and middle-income countries. In China, imatinib for CML costs approximately US $46,000 per year [5] while in India, trastuzumab currently costs approximately $US1,000 per month [6]. These prices are both well above the median salary in these countries, and because these countries are struggling to establish systems for universal health coverage [7] these medicines are currently available to only the wealthiest groups.

While not all PMs targeted to particular diseases are expensive, or as morally and politically contentious as are targeted cancer therapies (and while, of course, these kinds of PMs are not the only health interventions with potentially high opportunity costs), situations such as those described above raise unavoidable questions about population-level cost-effectiveness, value and opportunity costs. The funding of molecularly-targeted PMs also places payers in a difficult ethical and political position as they struggle to balance efficiency with equity—weighing up their desire to help small (sub) populations of patients against their desire to allocate resources fairly to whole populations.

## 4. Commercial Challenges of Personalized Medicine based on Molecular Diagnosis

Because the kind of PM we describe seeks to provide optimal treatment for individuals, based on their molecular profiles, it cannot, by definition, lead to the development of drugs to treat entire patient populations with apparently similar clinical conditions. PM of this kind thus challenges the traditional approach to drug development, where pharmaceutical companies have historically tried to develop drugs to meet a sizable “volume of need” associated with common illnesses such as cardiovascular disease and asthma. Because of the societal burden of such diseases, payers have been motivated to purchase treatments for them, thus enabling companies to recoup development costs and make a profit, without the need for very expensive per patient treatments. These circumstances essentially describe the recipe for a “blockbuster” drug, as illustrated by the success of Pfizer’s *Lipitor* [1,8] a drug that costs payers very little per patient per year but which has earned billions of dollars internationally for the company. This example is in contrast to the cost of personalized medicines, which generally cost payers many thousands of dollars per patient per year. In this regard, personalized medicines raise similar commercial issues to those raised by “orphan” medicines used to treat rare diseases, where companies often charge large sums of money in order to make a profit.

Personalized medicines that are based on molecular diagnostics also focus more on identifiable pathological targets rather than physiological ones. This is of commercial significance because patients who commence physiologically targeted drugs, such as bronchodilators and steroids used to treat asthma or lipid lowering agents used to treat hypercholesterolemia, often remain on treatment for the rest of their lives, thereby contributing to their status as “blockbuster’” medicines. By comparison, pathologically targeted drugs generally may not need to be taken for extended periods of time, unless the disease concerned has progressed to the point of becoming intrinsically complex, highly evolved and molecularly heterogeneous, as with advanced cancer or HIV. However, the opportunity for longer-term maintenance therapy is also often limited due to the poor prognoses for patients with such diseases. There are additional exceptions, with some physiologically targeted medicines used only for short periods, e.g., analgesics.

However, if a disease is appropriately diagnosed and molecularly characterized, pathologically targeted therapies—and thus many personalized medicines—may only need to be taken for finite periods of time as they limit the diseases ability to metabolically and physiologically maintain itself. An example being antibiotics selected for the management of bacterial infections due to *in vitro* sensitivities against the known pathogen.

Whether treatment duration of pathologically targeted therapies are limited due to poor survival outlook or cure the opportunity to recoup investments into the development of pathologically targeted treatments is less. Thus, medicines of this type tend to be expensive in terms of their per patient costs.

## 5. Epistemological Challenges of PM based on Molecular Diagnosis

One of the reasons that PM based on molecular diagnosis is such a challenge for those developing and funding medicines is that it forces us to question contemporary biomedical views of evidence, including its generation and use in decision-making. In particular, this kind of PM challenges the primacy of the large phase 3 randomized controlled trial (RCT) (and by extension systematic reviews and meta-analyses) as the optimal means for generating evidence about safety and efficacy, and requires the consideration of more complex methods of analysis and more nuanced (and sometimes ambiguous) forms of data. In large measure this results from the co-dependent development, use and assessment of medicines and diagnostics and from the ethical imperative to allow crossover in trials of personalized medicines. In this regard molecularly-targeted PM is similar to other paradigm shifts, such as the move towards “learning organizations” that challenge traditional conceptions, both of evidence and the ethics of generating evidence.

### 5.1. Challenges of Developing both Medicines and Co-Dependent Diagnostics

One advantage of prospectively designed RCTs is that they control for a number of known and unknown biases that may influence study outcomes. This is achieved by randomization of patients into two or more treatment groups. One challenge with PM based on molecular diagnosis is that there are two rather than one intervention under assessment—both the treatment and the test for the relevant molecular biomarker. This, in turn, makes it particularly difficult to know what populations to select for comparison, and it may also lead to a requirement for double randomization as a way of evaluating the overall benefit or impact of taking a PM and co-dependent treatment strategy.

#### 5.1.1. Population Selection

During early drug development, it can be difficult to know what populations to select for clinical trials. This is a particularly salient and complex issue in the testing of pathologically targeted therapies with the potential to become molecularly-targeted PMs. In clinical trials involving new physiologically-targeted drugs, it is generally fair to assume that all patients have the potential to respond to a new drug by virtue of them all possessing the same physiological target. This also means that any patient with the condition of interest could reasonably be included in a control group receiving an already proven physiologically targeted agent. In contrast, it may be much more difficult to establish what group of patients a pathologically-targeted PM should be tested against.

Trials of trastuzumab for breast cancer illustrate this problem. On one level, it would seem most appropriate to measure the benefit of trastuzumab *versus* standard care only in a population of patients who have breast cancer cells expressing very high levels of the receptor targeted by trastuzumab (HER-2), as this would provide information about efficacy and safety in the population who would be expected to benefit. Payers, however, may want to know how *all* patients fare in clinical trials, as all patients will require testing, a cost that will have to be factored into cost-effectiveness analyses. Yet, even if it is decided that costs of testing need to be factored into cost-effectiveness analyses (and trials need to be designed accordingly), this leaves open the question of what weighting should be applied to test and drug in terms of their costs when determining overall cost-effectiveness.

#### 5.1.2. Double Randomization

Merlin *et al.* have suggested two ways to overcome this “problem”, using a framework to help construct evidence for the cost effectiveness assessment of co-dependent health technologies [2,9], *i.e.*, drugs associated with a co-dependent diagnostic test. Both methods involve double randomization. 

The first method involves the randomization of patients into either a diagnostic tested or non-tested group. Patients within both groups are then further randomized to either receive a new investigative or standard treatment. Thus patients with a known diagnostic status may be randomized to receive either the new or standard of care treatment. Patients in the non-diagnostic tested group are also randomized to one of either treatment.

The second, similar, method also involves randomization to, or not to, diagnostic testing. However patients categorized by biomarker status are assigned treatments accordingly, with patients known to harbor the predictive biomarker receiving the pathologically targeted treatment under investigation while patients not harboring it are allocated the standard of care. Alternatively, patients randomized to the non-diagnostic testing arm are further randomized to either treatment.

Although Merlin *et al.* see these designs as providing “high-level evidence” [9], it is difficult to see how RCTs designed in these ways would be accepted by human research ethics committees (HREC). For if a diagnostic test used to predict disease response and determine therapy is incorporated into a study design, then this, in itself, suggests a strong scientific rationale exists for using it. This, in turn, may lead an HREC to question whether there is sufficient equipoise to justify randomizing patients to no testing, or to view the treatment of patients with a known diagnostic status inappropriate if there is a lack of scientific justification for targeted therapies they may be assigned to. It would be unacceptable, for example, for the economic assessment of HIV testing and drug treatment to be assessed in this way, were it presented today as a potential new management paradigm. An alternative approach to the testing of “co-dependent” technologies, that provides a balance between ethical imperatives and the need for scientific rigor, is therefore needed.

One such approach might be to make use of historical or contemporary biomarker-defined patient cohorts from outside of clinical studies [2,10]. However this requires access to data collections and good quality bio-specimens linked with patient outcomes. These cohorts would also need to be matched as closely as possible to those entered into an RCT used in the evaluation of a drug’s relative efficacy and safety. Furthermore, from an ethical perspective, there would need to have been appropriately obtained patient consent that provides sufficient scope for research into known and future (but unknown at the time of consent) links between biomarkers and outcomes.

### 5.2. Challenges in Demonstrating Value of Targeted Therapies

A second epistemological challenge that arises with particular significance in the context of PMs based on molecular diagnosis is that it can be difficult to demonstrate survival benefit because of the ethical requirement to allow crossover [4]. This issue is particularly salient in the development of PMs, with their pathological targeting, especially when the control or standard of care is a physiologically targeted agent. In addition, knowledge of the pathologic pathway and mode of action of treatment together with emerging evidence from other studies showing evidence of benefit make for more compelling arguments to crossover, raising additional ethical issues for investigators and those wishing to interpret evidence for overall survival. The development of imatinib for the treatment of chronic myeloid leukemia (CML) illustrates this difficulty.

The mainstays of therapy for CML had, up until the late 90s, been chemotherapy and bone marrow transplantation. Imatinib was developed with the knowledge that a dysfunctional enzyme, manufactured as a consequence of a mutation caused by the fusion of two separate regions of DNA (the “Philadelphia Chromosome”), was at the heart of the proliferative signaling cascade that leads to CML [3,11]. Despite apparently impressive responses to imatinib observed in the clinical setting [4,12], trials of imatinib were challenged in their ability to demonstrate the impact of treatment on patient survival. While this may have suggested that clinical impressions were somehow flawed, it was also possible that survival benefits in clinical trials were obscured by the fact that trials were not blinded, and patients crossed over from one arm to another in a non-random manner.

To explain further, blinding of studies where one arm was chemotherapy was not possible and, because of this, patients with progressive disease who were not initially randomized to imatinib would, understandably, have been keen to commence treatment as soon as possible. In other words, early indications from interim study analysis and the lack of alternative treatment options created an ethical imperative to enable access to imatinib. The result was dilution of the overall survival signal as patients ceased chemotherapy and crossed over to imatinib therapy, either whilst still on trial or through compassionate access programs opened by the manufacturer Novartis [5,13].

While it is difficult to imagine a situation in which it would be ethically acceptable to force patients to remain on standard chemotherapy, payers still tend to base their decisions on survival endpoints demonstrated in RCTs. While reliance on such “hard” historical endpoints is understandable, the possibility that this may lead to a failure to recognize real survival benefits is a source of major frustration to clinicians, patients and pharmaceutical sponsors seeking regulatory and reimbursement approvals [6,14]. In Australia, for example, the drug company, Roche have taken the decision not to seek public reimbursement of their BRAF targeted melanoma drug vemurafenib following its rejection twice by the Pharmaceutical Benefits Advisory Committee, despite melanoma being considered as “Australia’s” cancer, and despite vemurafenib being funded in other countries.

## 6. Conclusions

The realization of opportunities for healthcare provided by greater uptake of PM as defined in this article remains largely unfulfilled. While at first glance this might seem surprising given the enormous investment in the development of PM based on molecular diagnosis, and its congruence with the goal of patient-centered care, it is clear that PM of this kind also poses a number of ethical, economic and evidentiary challenges. Indeed, the barriers to uptake of PM are likely to be as much “cultural” as they are ethical, economic or scientific in the sense that they threaten familiar social norms and values. There is nothing new about this—it has long been recognized that paradigm shifts that we come take for granted (such as the move towards “evidence-based medicine”) come about only through sustained efforts on the part of advocates to shift existing epistemic, moral and socio-political norms and values [7,15]. Understanding and accepting that ethical, economic and epistemic barriers exist, and that culture change will be required, must be the first steps towards promoting uptake of molecularly-targeted PM. The responsibility for this resides with those able to bring about change, including the pharmaceutical industry, its regulators and those paying for medicines. 
